# Assembly and analysis of a *qingke* reference genome demonstrate its close genetic relation to modern cultivated barley

**DOI:** 10.1111/pbi.12826

**Published:** 2017-10-05

**Authors:** Fei Dai, Xiaolei Wang, Xiao‐Qi Zhang, Zhonghua Chen, Eviatar Nevo, Gulei Jin, Dezhi Wu, Chengdao Li, Guoping Zhang

**Affiliations:** ^1^ Department of Agronomy Zhejiang Key Lab of Crop Germplasm Zhejiang University Hangzhou China; ^2^ Western Barley Genetics Alliance Western Australian State Agricultural Biotechnology Centre School of Veterinary and Life Sciences Murdoch University Perth WA Australia; ^3^ School of Science and Health Western Sydney University Penrith NSW Australia; ^4^ Institute of Evolution University of Haifa Haifa Israel

**Keywords:** genomic contribution, hulless barley, reference genome, RNA‐sequencing, third‐generation sequencing

## Abstract

*Qingke*, the local name of hulless barley in the Tibetan Plateau, is a staple food for Tibetans. The availability of its reference genome sequences could be useful for studies on breeding and molecular evolution. Taking advantage of the third‐generation sequencer (PacBio), we *de novo* assembled a 4.84‐Gb genome sequence of *qingke, cv*. Zangqing320 and anchored a 4.59‐Gb sequence to seven chromosomes. Of the 46,787 annotated ‘high‐confidence’ genes, 31 564 were validated by RNA‐sequencing data of 39 wild and cultivated barley genotypes with wide genetic diversity, and the results were also confirmed by nonredundant protein database from NCBI. As some gaps in the reference genome of Morex were covered in the reference genome of Zangqing320 by PacBio reads, we believe that the Zangqing320 genome provides the useful supplements for the Morex genome. Using the *qingke* genome as a reference, we conducted a genome comparison, revealing a close genetic relationship between a hulled barley (*cv*. Morex) and a hulless barley (*cv*. Zangqing320), which is strongly supported by the low‐diversity regions in the two genomes. Considering the origin of Morex from its breeding pedigree, we then demonstrated a close genomic relationship between modern cultivated barley and *qingke*. Given this genomic relationship and the large genetic diversity between *qingke* and modern cultivated barley, we propose that *qingke* could provide elite genes for barley improvement.

## Introduction

Barley (*Hordeums vulgare* L.) ranks the fourth largest cereal in planting area worldwide and is one of the earliest domesticated crops (Purugganan and Fuller, 2009). As an excellent model plant for understanding agronomic and physiological responses to climate change (Dawson *et al*., 2015), barley may provide insight into the mechanisms of abiotic stress adaptation and tolerance in cereal crops. The Tibetan Plateau is the home of *qingke*, the local name of hulless barley, which has been used as a staple food of Tibetans for thousands of years (Xu, 1982). The Near East Fertile Crescent is well‐recognized as an original site of wild barley (*Hordeum spontaneum* L.) and a major domestication centre of cultivated barley (Nevo, 2006; Zohary *et al*., 2012), but increasing evidence supports a theory of polyphyletic origin of cultivated barley (Azhaguvel and Komatsuda, 2007; Dai *et al*., 2012, Dai *et al*., 2014; Molina‐Cano *et al*., 2005; Morrell and Clegg, 2007).

Barley is an annual diploid grass species with a large haploid genome of 5.1 Gb which contains a high abundance of repetitive elements (Mayer *et al*., 2012). An accurate genome sequence is a prerequisite for molecular breeding and a deeper understanding of its evolution. The high‐throughput sequencing capacity of second‐generation sequencers enables more timely, and cost‐effective assembly of diploid plant genomes (Michael and VanBuren, 2015), but can be difficult to assemble a high‐quality reference genome sequence for plants like barley with large genomes (Zeng *et al*., 2015). The Pacific Biosciences real‐time single‐molecule (PacBio RS) third‐generation sequencer has the potential to increase read lengths dramatically and thus improve *de novo* genome assembly (Gordon *et al*., 2016; Rasko *et al*., 2011); the PacBio RS can generate kilo‐base long reads to fill gaps in scaffolds assembled from second‐generation sequencers.

Recently, a 3.48‐Gb (anchored to seven chromosomes) draft genome of hulless barley, *cv*. Lasa Goumang has been reported (Zeng *et al*., 2015), but this genome is not yet available publicly. As a member of the International Barley Genome Sequencing Consortium, we have access to the latest genome sequence data set of hulled barley *cv*. Morex (Mascher *et al*., 2017), which has been used to conduct a comparative genomic study. In this study, we aimed to (1) construct a complementary reference genome sequence of hulless barley *cv*. Zangqing320 using a leading sequencing strategy to combine the whole‐genome shotgun method with PacBio third‐generation sequencing and (2) demonstrate the genomic relationship between *qingke* and modern cultivated barley.

## Results

### Sequencing and *de novo* assembly of a reference genome of hulless barley *cv*. Zangqing320

A whole‐genome shotgun method combined with the third‐generation sequencing strategy was used to resequence, and *de novo* assemble the reference genome of hulless barley *cv*. Zangqing320 from the Tibetan Plateau. We generated 269.81 Gb of Illumina sequence data including 32.37, 84.63 and 17.12 Gb from libraries of 250, 300 and 500 bp, respectively, and 117.40 and 18.29 Gb from the 2‐ and 6‐kb mate‐pair libraries, respectively (Table S1). Moreover, PacBio third‐generation sequencers generated much longer reads with an average subread length of 3 kb (Table S1).

**Figure 1:**
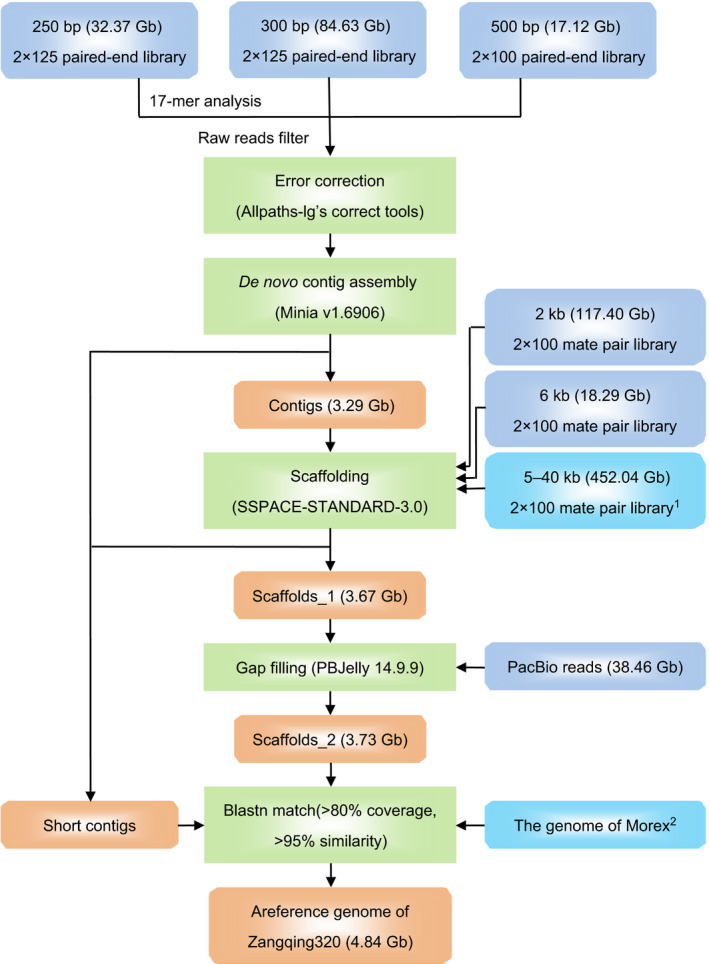
Schematic workflow for genome assembly of a hulless barley, cv. Zangqing320 from the Tibetan Plateau. Data from the current study are dark blue, data adapted from previous studies are light blue, operation processes are green, and assembly results are orange. ^1^ Data from Zeng *et al*. (2015). ^2^ The reference genome of Morex from Mascher *et al*. (2017) .

*De novo* contig assembly was performed after filtration and error correction of raw reads (Figure 1), generating 632,382 contigs (3.29 Gb) with 44.28% GC content and an N50 length of 5.94 kb (Table 1). Mate‐pair reads of the 2‐ and 6‐kb libraries, and those of 5‐ to 40‐kb libraries (SRR1804516‐SRR1804532) adapted from Zeng *et al*. (2015), were used to link contigs into scaffolds, resulting in 104,997 scaffolds (scaffolds_1, 3.67 Gb) with 103,894 gaps, 9.32% N content, 44.28% GC content and an N50 length of 173.83 kb (Table 1, Figure 1). Gaps in scaffolds_1 were filled with 38.46 Gb PacBio reads, resulting in scaffolds_2 (3.73 Gb) with 89 678 gaps and 91 879 scaffolds (Table 1, Figure 1). Using the framework of reference genome sequence of hulled barley *cv*. Morex (Mascher *et al*., 2017), we constructed a 4.84‐Gb reference genome of hulless barley *cv*. Zangqing320 for research on molecular evolution, domestication, gene cloning and functional analysis, and molecular breeding in barley (available at http://www.ibgs.zju.edu.cn/ZJU_barleygenome.htm), with 8.0% N and 44.44% GC content, and 4.59 Gb anchored to seven chromosomes (Table 1, Figure 1).

**Table 1:** Statistics of the reference genome of a hulless barley, *cv*. Zangqing320 from the Tibetan Plateau

Genomic statistics	Contigs	Scaffolds_1*	Scaffolds_2†	Genome
Total sequence length	3.29 Gb	3.67 Gb	3.73 Gb	4.84 Gb
Number of contig/scaffold/chromosome	632 382	104 997	91 879	7‡
Longest contig/scaffold/chromosome	298.21 kb	2.69 Mb	2.69 Mb	768.77 Mb
N50 length	5.94 kb	173.83 kb	171.11 kb	–
Average length	5.21 kb	35.00 kb	40.56 kb	604.76 Mb
Gap number	0	103,894	89,678	1,172,646
N content (%)	0.00	9.32	7.74	8.00
GC content (%)	44.28	44.28	43.64	44.44

*Scaffolds_1 were assembled from second‐generation contigs, gaps in which were filled with PacBio RS reads and resulted in ^†^scaffolds_2 refer to Figure 1. ^‡^Sequence not anchored to chromosomes 1H–7H were named as chrUn.

### Genome annotation identifies a large number of high‐confidence genes

We conducted a genome annotation using 1.17 billion RNA‐sequencing (RNA‐Seq) reads from seedling leaves of 39 wild and cultivated barley genotypes with wide genetic diversity (Table S2). After removing genes shorter than 300 bp or longer than 20 kb, we identified 38 085 genes in the Zangqing320 genome. Based on annotated genes in the assembly genome of Morex (version: 082214v1, http://plants.ensembl.org/index.html) and homolog support of nonredundant (nr) protein database from the National Center for Biological Information (NCBI) using Blast2GO, 31 564 genes in the reference genomes of Zangqing320 were deemed ‘high‐confidence’ genes.

As all of the RNA‐Seq data used for gene prediction were obtained from leaf tissues at the vegetative stage, we further performed *de novo* gene prediction to generate a complete genome annotation file. A total of 197 063 genes were *de novo* predicted, including 32 482 genes with homolog support from the NCBI nr protein database. Excluding genes encoding protein which less than 100 amino acids or containing Ns, 30 539 *de novo* predicted genes supported from the nr protein database were compared to the 31 564 RNA‐Seq predicted ‘high‐confidence’ genes to identify common genes. After excluding those common genes, 15 223 *de novo* predicted genes were added, to generate a genome annotation file of Zangqing320 with 46 787 annotated genes.

### Genome comparison reveals a close genetic relation between Morex and Zangqing320 in genome structure

**Figure 2:**
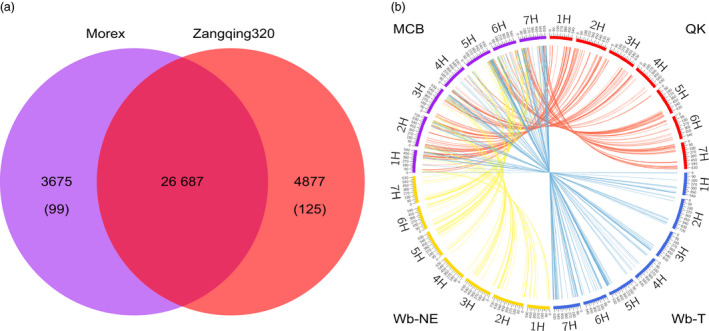
Genome comparison (a) and genomic similarity analysis (b). (a) Common and specific annotated genes in the reference genome of Morex and Zangqing320. Private genes (shown in brackets) had no homologous gene in the other genotype. (b) Analysis of genomic similarities between the modern cultivated barley group and the other three barley groups. The outer track of the circos diagram shows the seven chromosomes (1H–7H) of barley in each of the four groups: purple, modern cultivated barley (MCB); red, hulless barley from the Tibetan Plateau (QK); blue, wild barley from the Tibetan Plateau (Wb‐T); yellow, wild barley from the Near East (Wb‐NE). The number on each chromosome indicates the genomic position on the genome of Zangqing320 (Mb). Similar blocks are connected with lines, and each line represents one unique window (500 kb) of the genome with the highest similarity between MCB and the other three barley groups.

**Figure 3:**
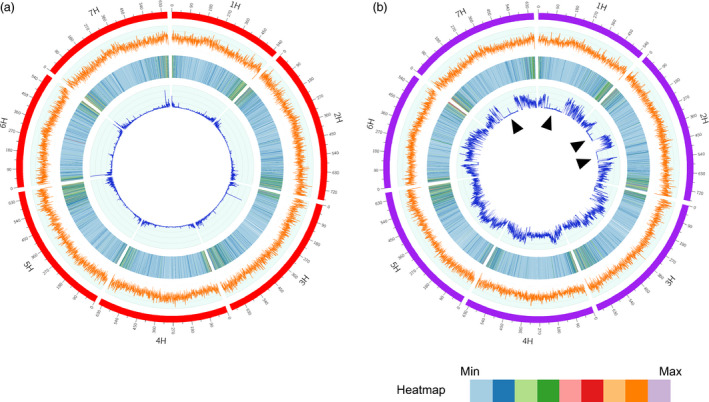
Genome characterization of Zangqing320 (a) and Morex (b). The four tracks from outside to inside are chromosomes (red for Zangqing320, purple for Morex), CpG island density (orange), gene density (heatmap), and single nucleotide variant (SNV) density (dark blue). The densities of the CpG island, gene and SNV are represented by the number in nonoverlapping 500‐kb windows. SNVs in (a) show the 125,157 SNV data set with homozygous loci and no missing data called by mapping RNA‐Seq reads of the 39 barley accessions to the reference genome of hulless barley *cv*. Zangqing320, while SNVs in (b) are called after mapping reads of Zangqing320 to the genome of Morex. Black triangles in the innermost track of (b) refer to genomic regions with low genetic diversity between Morex and Zangqing320.

To compare the genome similarity between Morex and Zangqing 320, we annotated the genome of Morex with the RNA‐Seq data of 39 barley genotypes and identified 30 362 ‘high‐confidence’ genes. We then compared sequences of the RNA‐Seq predicted ‘high‐confidence’ genes in Morex and Zangqing320 using tblastx. We found 26 687 common genes along with 3675 and 4877 potential private genes in Morex and Zangqing320, respectively (Figure 2a). Private genes refer to those detected only in Morex or Zangqing320. Further comparison of potential private genes using tblastx excluded similar genes and detected 99 and 125 private genes in Morex and Zangqing320, respectively (Figure 2a). The low number of private genes indicates a close genetic relation between Morex and Zangqing320 in genome structure. The position of single nucleotide variants (SNVs) and CpG island densities (Figure 3a,b) also support this finding. For example, the seven chromosomes of Morex and Zangqing320 contained 206 049 and 206 152 CpG islands, respectively (Table S3), with the highest density on chromosome 3H in both genotypes.

### Whole‐genome similarity analysis demonstrates a close genomic relationship between modern cultivated barley and *qingke*

**Figure 4:**
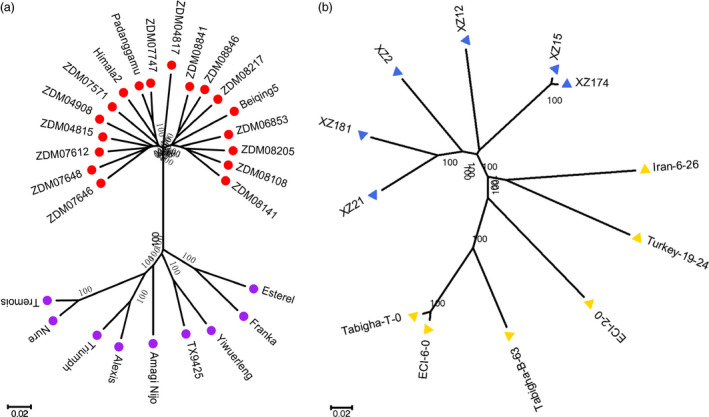
Phylogenetic trees of cultivated barley (a) and wild barley (b) based on the 125 157 SNVs data set randomly distributed in the seven barley chromosomes. The neighbour‐joining method was used with 1000 bootstraps. Branches marked by red dots, purple dots, blue triangles and yellow triangles represent hulless barley from the Tibetan Plateau, modern cultivated barley, wild barley from the Tibetan Plateau, and wild barley from the Near East, respectively.

In addition to genome comparison, we further analysed the genomic similarity between modern cultivated barley and *qingke* using the method in our previous study (Dai *et al*., 2014) with one additional cultivar and 15 *qingke* accessions. Aligning RNA‐Seq reads in each of the 39 samples to the Zangqing320 genome, we identified 1 148 006 SNVs on the seven chromosomes (Tables S2 and S3). Wide genetic variation was detected in both wild (including those from the Tibetan Plateau and the Near East) and cultivated barley genotypes, with an average of 147 797 and 126 955 SNVs, respectively (Table S2). However, in 18 *qingke* accessions, only an average of 109 050 SNVs was detected (Table S2). To avoid unreliable inferences caused by missing and multiple variation sites, we constructed a data set containing 125 157 SNVs with homozygous loci and no missing data in 39 samples to perform genomic similarity analysis (Figure 3a). We divided the 39 barley genotypes into four groups according to phylogenetic trees (Figures 4 and S1), population structure (Figure S2), information on collection sites (Table S2) and the results of our previous study (Dai *et al*., 2014): (1) modern cultivated barley (MCB, nine modern barley cultivars), (2) QK (18 *qingke* accessions), (3) wild barley from the Tibetan Plateau (Wb‐T, including XZ2, XZ12, XZ15, XZ21, XZ174 and XZ181) and (4) wild barley from the Near East (Wb‐NE, including ECI‐2‐0, ECI‐6‐0, Tabigha‐B‐63, Tabigha‐T‐0, Iran‐6‐26 and Turkey‐19‐24) (Table S2).

As described in previous studies (Dai *et al*., 2014; Rubin *et al*., 2010), each group of barley genotypes was combined as a gene pool. With 500‐kb windows and 250‐kb overlapping slide windows along the Zangqing320 genome, 714 similar genetic windows containing 35 665 SNVs met the selection criteria, accounting for 5.9% of the genome. The genomic similarities between MCB and QK, and Wb‐T and Wb‐NE were visualized with a circos diagram (Figure 2b). As expected, the two cultivated barley groups, MCB and QK, had a tight genomic relationship (Figure 2b, Table S4). Only 154 similar genetic windows were identified between MCB and Wb‐NE, while 334 and 226 unique genetic windows were detected between MCB and QK, and MCB and Wb‐T, respectively (Table S4). The genomic similarity of MCB and the other three barley groups was calculated based on the total length of unique genomic windows. MCB and QK had a high genomic similarity value on chromosomes 1H, 2H, 3H and 4H, being 60.67%, 59.63%, 55.56% and 49.18%, respectively (Table S4). The genomic similarity between MCB and Wb‐T was 41.98% on chromosome 6H (Table S4).

Moreover, to confirm our previous finding on *qingke*'s close genetic relationship with Tibetan wild barley, while different from modern cultivated barley (Dai *et al*., 2014), we performed genomic similarity analysis focusing on QK to determine the genomic relationships among the four barley groups. Wb‐T had the tightest genomic relationship with QK, followed by MCB and Wb‐NE (Figure S3a, Table S4), which also reflected the reliability of grouping. Among the wild barley groups, the genomic similarity between QK and Wb‐T was more than fivefold higher than that between QK and Wb‐NE (Figure S3b, Table S4). Obviously, *qingke* is closely associated with the Tibetan wild barley at the genome level.

### Selective sweep analysis reveals novel genes between modern cultivated barley and *qingke*

**Figure 5:**
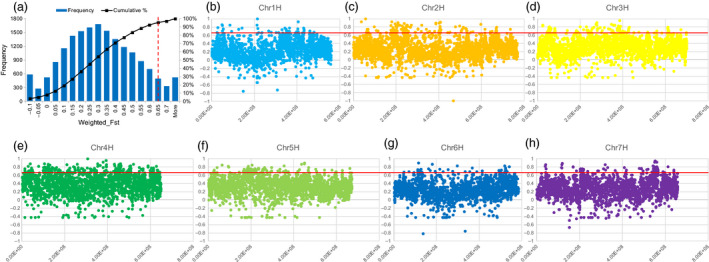
Distribution of windowed *F*_ST_ values between *qingke* (QK) and modern cultivated barley (MCB) across the whole genome (a) or along each chromosome (b, c, d, e, f, g, h) of Zangqing320. *F*_ST_ value was calculated in each 500‐kb region in 250‐kb steps. (a) The x‐axis indicates the value of *F*_ST_ and the y‐axis shows the frequency of *F*_ST_ value. The red dash vertical line (in panel a) indicates the threshold value which is chosen based on the distribution of all windowed *F*_ST_ . (b, c, d, e, f, g, h) X‐axis indicates the physical position of each chromosome and y‐axis shows the cumulative percentage. The red horizontal line indicates the threshold value which is chosen based on the distribution of all windowed *F*_ST_ .

Selective sweep analysis, a major method used to detect environmental or natural selection signatures, was performed between MCB and QK to detect the genome regions under selective sweeps affected by the unique growth environment in the Tibetan Plateau. Fixation index (*F*_ST_) was used to measure genetic differentiation of the two barley groups, and genome regions with *F*_ST_ > 0.65 (about 5% top *F*_ST_ windows) between MCB and QK were determined as selective sweeps (Figure 5).

**Figure 6:**
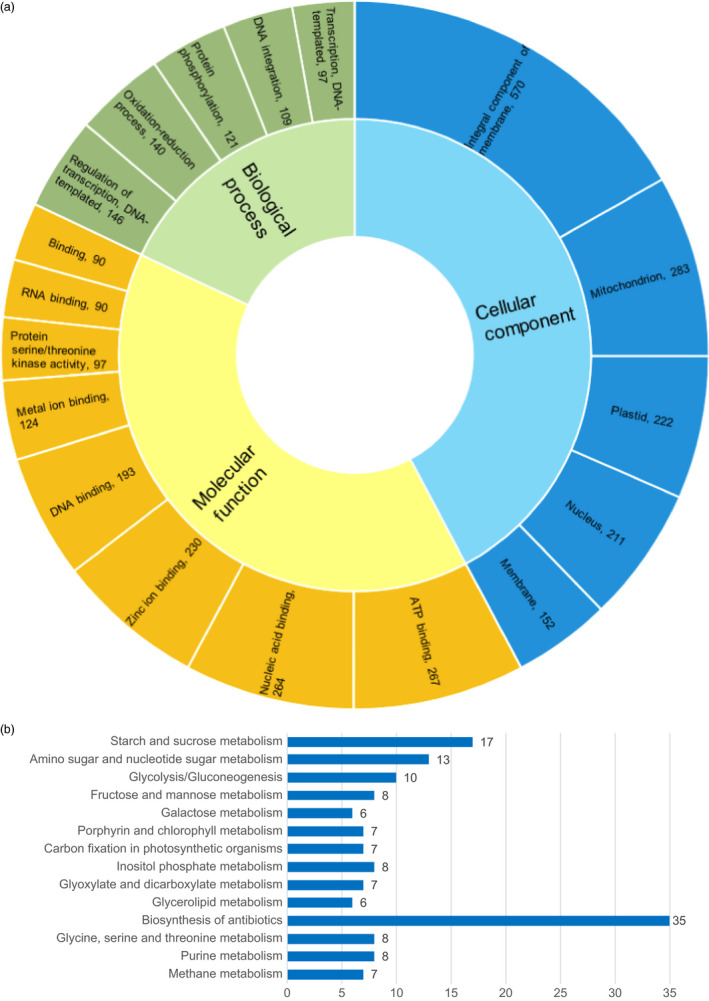
Top 1% Gene Ontology (GO) terms (a) and top 15% Kyoto Encyclopedia of Genes and Genomes (KEGG) pathways (b) analysis of the 2544 genes in selection sweep regions of qingke (QK) and modern cultivated barley (MCB). Numbers of genes with a certain GO term are listed after GO names separated by a comma (a), while those for the KEGG pathway are marked on the right side of the bar (b).

The above analysis identified 7.25% of the genome sequence and 5.44% (2,544) of the annotated genes as involved in the selection (Figure 5, Data S1). Interestingly, up to 16.33% of chromosome 4H was under selective sweeps (Figure 5). Gene Ontology (GO) analysis of the 2,544 genes under selective sweeps revealed that ‘integral component of membrane’, ‘ATP binding’ and ‘regulation of transcription, DNA‐templated’ were the most enriched terms in cellular component, molecular function, and biological process, respectively, followed by ‘mitochondrion’, ‘nucleic acid binding’, and ‘oxidation‐reduction process’, and then ‘plastid’, ‘zinc ion binding’ and ‘protein phosphorylation’ (Figure 6a). In addition, the Kyoto Encyclopedia of Genes and Genomes (KEGG) analysis showed that they are mainly enriched in pathways associated with carbohydrate metabolism (e.g. starch and sucrose metabolism, amino sugar and nucleotide sugar metabolism, glycolysis/gluconeogenesis, fructose and mannose metabolism, and galactose metabolism), photosynthesis (e.g. porphyrin and chlorophyll metabolism and carbon fixation in photosynthetic organisms) and glycerolipid metabolism (Figure 6b).

## Discussion

### Third‐generation sequencing provides a high‐quality reference genome of hulless barley *cv*. Zangqing320

**Figure 7:**
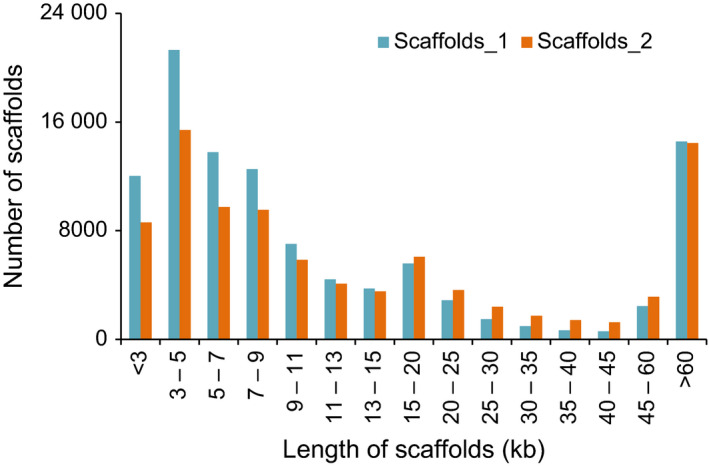
Length distribution of sequences in scaffolds_1 and scaffolds_2 of Zangqing320. The x‐axis indicates the length of scaffolds (kb), and the y‐axis shows the number of scaffolds. Scaffolds_1 were assembled from second‐generation contigs; the gaps were filled with PacBio RS reads and resulted in scaffolds_2 (refer to Figure 1).

The read length of second‐generation sequencers could not span the long, repetitive sequences that make up more than 80% of the barley genome (Mayer *et al*., 2012; Wicker *et al*., 2009). Third‐generation sequencers, however, have the potential to dramatically improve the read length for *de novo* genome assembly (Gordon *et al*., 2016; Rasko *et al*., 2011). In the current study, we employed the PacBio RS sequencer to cross over the long, repetitive sequences in the barley genome. The PacBio RS reads fill the gaps in scaffolds *de novo* assembled from second‐generation reads, closing 13.68% of the gaps, eliminating 15.77% of the Ns, and increasing the average scaffold length in scaffolds_1 by 15.80%, thus resulting in scaffolds_2 (Table 1, Figure 1). Comparing scaffolds_1 and scaffolds_2, the latter had fewer scaffolds shorter than 15 kb and more scaffolds ranging from 15 to 60 kb (Figure 7). The number of scaffolds longer than 60 kb declined slightly in scaffolds_2, possibly because the gaps with excessive Ns in scaffolds_1, introduced from the mate‐pair reads, were filled with shorter‐than‐expected sequences. Taking advantage of the PacBio RS reads, we generated a high‐quality reference genome of hulless barley *cv*. Zangqing320 that was 1.11 Gb larger than the recently reported 3.48‐Gb (anchored to seven chromosomes) genome of hulless barley *cv*. Lasa Goumang (Zeng *et al*., 2015). The reference genome of Zangqing320 showed comparable length to that of hulled barley *cv*. Morex. As some gaps in the reference genome of Morex were covered in the reference genome of Zangqing320 by third‐generation PacBio reads, we believe both reference genomes would be useful for barley breeding and genetic research in Triticeae.

### Genomic‐based analysis revealed genetic diversity of *qingke*

The genome structure of Morex and Zangqing320 indicated a close genetic relationship. However, we detected large genetic diversity in the two sequenced reference barley genomes. By mapping the in‐depth resequencing reads of Zangqing320 to the reference genome of Morex, 6 053 445 SNVs were detected on the seven chromosomes (Figure 3b, Table S3). The difference was also reflected in the 3675 and 4877 specific annotated genes in the reference genome of Morex and Zangqing320, respectively. Gene Ontology (GO) analysis of specific genes indicated that 19.04% and 44.92% GO terms are found exclusively in Morex and Zangqing320, respectively, although the top 1% GO terms are highly identical (Figure S4). For instance, enriched unique GO terms of specific genes in Morex were associated with salicylic acid signalling, kinase, and membrane‐involution autophagy and transport, while those in Zangqing320 were relevant to cytokinin signalling, phosphatase, cell division, polysaccharide catabolic, transcription and post‐translational modification (Figure S5). Surprisingly, 19 548 975 and 23 394 190 SNVs were detected on seven chromosomes by mapping the in‐depth resequencing reads of barley cultivars Igri and Barke to the reference genome of hulless barley *cv*. Zangqing320, respectively. The vast number of SNVs may be due to the higher completion of the reference genome of hulless barley *cv*. Zangqing320. The results also indicated that, compared with *cv*. Morex, *cv*. Zangqing320 has a greater genetic difference between *cv*. Igri and *cv*. Barke.

In addition to the genomic sequence analysis, we conducted a comparative population analysis at the transcriptome level and found a significant genetic difference between modern cultivated barley and *qingke*. About 1.73% of unique SNVs in the 125 157 SNVs data set were detected in QK, which will be a valuable genetic resource for *qingke* utilization and gene mining. According to the selective sweep analysis and gene annotation, we detected nine, seven, and five genes under selective sweeps encoding peroxidase, TRANSPARENT TESTA 12, and ultraviolet‐B receptor UVR8, respectively (Data S1). It is reported that *qingke* is well adapted to harsh environments which include the strong ultraviolet light and unpredictable diurnal temperature fluctuations in the Tibetan Plateau (Xu, 1982). Thus, these results should be useful for future barley improvement.

### *Qingke* genome may have contributed to modern cultivated barley

In our previous study, we proposed that *qingke* probably existed in an early stage of domestication (Dai *et al*., 2014). Here, we provide further evidence to support the idea that *qingke* shares a large proportion of its genome with modern cultivated barley. Gene comparison among cereal species can identify genomic regions that are highly conserved or rapidly evolving, providing deeper insight into genome evolution, speciation and domestication (Matsumoto *et al*., 2005). Interestingly, there were four genomic regions larger than 100 Megabase (Mb) on chromosomes 1H (74.5–305.0 Mb), 2H (106.5–258.0 Mb and 399.5–500.5 Mb) and 7H (222.0–427.0 Mb), showing very low genetic diversity between Zangqing320 and Morex (Figure 3b). In contrast, the conserved regions on chromosomes 1H and 2H were much narrower between Zangqing320 and Igri (1H: 101.0–272.0 Mb, 2H: 128.0–174.0 Mb and 409.5–438.0 Mb), and between Zangqing320 and Barke (1H: 101.0–315.0 Mb, 2H: 121.0–174.0 Mb and 409.5–496.0 Mb), while the conserved region of 205 Mb on 7H between Zangqing320 and Morex was not detected between Zangqing320 and Igri or Barke (Figure S6).

It is well documented that barley used for breeding and cultivation in North America initially came from germplasm introduced mainly from Europe and East Asia (Horsley and Harvey, 2011). For instance, the most significant genetic donor of Morex is Manchuria, a six‐rowed barley landrace introduced from Northeast China in the early 1900s, where part of its genome may be similar to that of Tibetan hulless barley. However, from the pedigree information of Igri and Barke that were released in Europe (Barley Pedigree Online, http://genbank.vurv.cz/barley/pedigree/pedigree.asp), the two cultivars appear to have no close genetic link with Zangqing320. Thus, given the breeding history of modern cultivated barley (Ullrich, 2011) and the current data, we hypothesize that *qingke* or landraces from China have made a substantial genomic contribution to modern cultivated barley.

## Experimental procedures

### Plant materials

A hulless barley cultivar Zangqing320 from the Tibetan Plateau was used in this study for in‐depth genome resequencing and *de novo* assembly. Zangqing320 was derived from a cross between a breeding line 7323 (derived from a cross between Zangqing 334 ×  Lashabaiqingke) and *cv*. Zangqing 7239. Fifteen *qingke* accessions (Table S2) and a malting barley cultivar, Triumph, were used to conduct RNA‐Seq at the seedling stage for SNV analysis. In addition, RNA‐Seq data of 12 wild and 11 cultivated barley genotypes (including three *qingke* genotypes) (Table S2) from our previous study were used for SNV analysis (Dai *et al*., 2014; Wang *et al*., 2016).

### Genome sequencing

Genomic DNA was isolated from leaves of Zangqing320 at the seedling stage. For each short‐insert size library construction, 5 μg of DNA was fragmented, end‐repaired, size‐selected at 250, 300 and 500 base pairs (bp) on agarose gels, and ligated to Illumina PCR‐free paired‐end adapters. For each large‐insert size mate‐pair library, 20–60 μg of genomic DNA was sheared to the desired insert size using nebulization for 2 kb or HydroShear for 6 kb. DNA fragments were biotin‐labelled, size‐selected and circularized. Circular DNA molecules were sheared with Adaptive Focused Acoustic (Covaris, Woburn, MA) to an average size of 450 bp. Biotinylated fragments were purified on magnetic beads (Invitrogen, Carlsbad, CA) and used to construct libraries. DNA paired‐end libraries of 250 and 300 bp were sequenced using Illumina HiSeq 2500 sequencing platform (Illumina, San Diego, CA). DNA paired‐end libraries of 500 bp were sequenced using Illumina NextSeq 500 platform (Illumina). DNA mate‐pair libraries of 2 and 6 kb were sequenced using Illumina HiSeq 2000 platform (Illumina) (Table S1).

Single‐molecule real‐time (SMRT) bell genomic libraries and SMRT sequence data (P4C2 chemistry, RSII platform) were generated and sequenced using a Pacific Biosciences (PacBio) SMRT sequencer (Pacific Biosciences, Menlo Park, CA) (Eid *et al*., 2009). The raw data reported in this study have been deposited in the National Center for Biotechnology Information Sequence Read Archive (www.ncbi.nlm.nih.gov).

### Genome assembly

Raw reads were stringently filtered by removing: (1) reads containing more than 2% Ns or with poly‐A structure, (2) reads containing 40% and 60% or more low‐quality bases for short‐insert and large‐insert size libraries, respectively, (3) adapter‐polluted reads, (4) reads overlapping between read1 and read2, and (5) PCR‐duplicated reads. The 17‐mer analysis was performed using the sequencing data from the 250 bp 2 × 125 paired‐end library, indicating that the peak frequency of K depth was about 21. Error correcting was undertaken using Allpaths‐lg's tools (Gnerre *et al*., 2011).

*De novo* contig assembly was conducted by Minia v1.6906 (Chikhi and Rizk, 2013). SSPACE‐STANDARD‐3.0 (Boetzer *et al*., 2011) was used to link contigs into scaffolds with mate‐pair reads. Gaps in scaffolds_1 were filled using PBJelly 14.9.9 (English *et al*., 2012) with PacBio reads. Based on the reference genome of Morex (Mascher *et al*., 2017), blastn was performed with the definition standards of ‘more than 80% coverage and more than 95% similarity’ to anchor scaffolds and short contigs not making up scaffolds onto chromosomes (Figure 1).

### Genome annotation and comparison between *cv*. Morex and *cv*. Zangqing320

After removing all adaptor sequences and low‐quality reads (Q < 30 and length <50 bp), RNA‐Seq reads of 39 genotypes were mapped to the genomes of Morex and Zangqing320 using Hisat2 v2.0.4 (Kim *et al*., 2015) with parameters of ‘–dta –score‐min L,0,‐0.8 ‐5 10 ‐3 10’. SAM files created by Hisat2 were cleaned by CleanSam.jar program of picard‐tools‐1.119 (http://picard.sourceforge.net) and converted to BAM files using SAM‐tools (Li *et al*., 2009). After sorting and correcting with SAM‐tools (Li *et al*., 2009), BAM files were submitted to Stringtie v1.2.3 (Pertea *et al*., 2015). Transcripts were reconstructed and then merged using Stringtie v1.2.3 (Pertea *et al*., 2015) with default parameters.

After removing genes shorter than 300 bp and longer than 20 kb, gene sequences constructed by Stringtie were aligned with annotated gene sequences in the assembly genome of *cv*. Morex (version: 082214v1, http://plants.ensembl.org/index.html) using blastn of Blast v2.2.28 (Altschul *et al*., 1990) with parameters of ‘‐max_target_seqs 1 ‐evalue 1E–5^’^. The remaining gene sequences constructed by Stringtie were submitted to the blastx program of Blast2GO v3.0 (Conesa *et al*., 2005) with an e‐value of 1e^−3^, and a blast DB of nonredundant protein sequences (nr). Based on the homolog support from blastn with genes of Morex (version: 082214v1) and the latter blastx with NCBI nr protein database, ‘high‐confidence’ genes of Morex and Zangqing320 predicted from RNA‐Seq data were identified.

In addition to the ‘high‐confidence’ genes predicted from RNA‐Seq data, *de novo* gene prediction was conducted using Augustus V2.7 (Stanke *et al*., 2006) to generate a complete genome annotation file of Zangqing320. Protein sequences of *de novo* predicted genes were compared with the nr protein database using blastp (Altschul *et al*., 1990) with an e‐value of 1e^−5^. Using Blat (Kent, 2002) as well as gene position, *de novo* predicted genes were compared with the 31 564 RNA‐Seq predicted ‘high‐confidence’ genes in the genome of Zangqing320. After removing redundant genes with similarity greater than 97% and match length longer than 200 bp, or with the same position on the genome, we combined these two data sets of predicted genes to produce a genome annotation file of Zangqing320.

To determine any private genes within Morex or Zangqing320, tblastx of Blast v2.2.28 (Altschul *et al*., 1990) was conducted between the 30 362 and 31 564 RNA‐Seq predicted ‘high‐confidence’ genes of Morex and Zangqing320 with the parameters ‘‐max_target_seqs 1 ‐evalue 1E–5^’^. CpG islands—DNA regions rich in CpG dinucleotides (Han *et al*., 2008)—were predicted using EMBOSS CpGPlot (http://www.ebi.ac.uk/emboss/cpgplot) (Larsen *et al*., 1992) with parameters of ‘‐window 10 000 ‐minlen 200’. Using BWA 0.7.5a‐r405 (Li and Durbin, 2010), genome reads of Zangqing320 were mapped to the reference genome of Morex (Mascher *et al*., 2017), while those of Igri (ERR125903) and Barke (ERP001450) were mapped to the reference genome of Zangqing320, SNVs were called with SAM‐tools mpileup and bcftools (Li *et al*., 2009). The raw SNVs were filtered with a mapping quality score ≥25 and reads coverage >2. The number of CpG islands, genes and SNVs in nonoverlapping 500‐kb windows across the genome sequence of Morex or Zangqing320 was calculated and then visualized using ClicO FS v2.0.0 (http://codoncloud.com:3000/) (Cheong *et al*., 2015).

### Transcriptome sequencing and SNV calling

The third fully expanded leaves were sampled from 15 *qingke* accessions and one hulled barley *cv*. Triumph for RNA‐Sequencing. Total RNA was extracted from a frozen leaf sample (~0.5 g) using TRIzol Reagent (Invitrogen). RNA was purified using RNeasy Mini Kit (Qiagen, Germantown, MD) and quality‐checked using the Agilent 2100 Bioanalyzer (Agilent Technologies, Palo Alto, CA). The RNA samples were frozen at −80 °C until required. Library construction and 2 × 150 bp paired‐ends sequencing on Illumina HiSeq Platform (Illumina) were performed as described by Dai *et al*. (2014).

After removal of adaptor sequences, empty reads and low‐quality reads (Q < 30 and length <50 bp) from raw reads, clean reads of the 39 samples were obtained. Hisat2 v2.0.4 (Kim *et al*., 2015) was used to map clean reads to the assembly genome of hulless barley *cv*. Zangqing320 with parameters of ‘–dta –score‐min L,0,‐0.8 ‐5 10 ‐3 10’. Raw SNVs and *indels* were called with SAM‐tools mpileup and bcftools (Li *et al*., 2009) and then filtered with mapping quality scores ≥25 and reads coverage >2.

### Phylogenetic, population structure and genomic similarity analysis

A data set of 125 157 SNVs with homozygous loci and no missing data in 39 samples were used to conduct phylogenetic and population structure analyses. The phylogenetic tree of the 39 accessions was constructed using MEGA 5.05 (Tamura *et al*., 2011) with neighbour‐joining methods (1000 bootstraps). The population structure was investigated using frappe1.1 (Tang *et al*., 2005) based on a maximum‐likelihood method, with 10 000 iterations and the number of clusters (K) set from 2 to 6. According to the results of the phylogenetic trees (Figures 4 and S1), population structure (Figure S2) analysis, information on collection sites (Table S2) and results from our previous study (Dai *et al*., 2014), we divided the 39 barley genotypes into four groups for genomic similarity analysis.

Genomic similarity analysis was performed according to Rubin *et al*. (2010) and Dai *et al*. (2014). Briefly, each SNV type with a known site was allocated to one of four groups (MCB, QK, Wb‐T and Wb‐NE) to construct four barley gene pools. We used 500‐kb windows and 250‐kb overlapping slide windows along the genome of Zangqing320. Unique genetic windows with the highest similarity were selected using the following criterion: ‘the number of SNVs in each window ≥25’ and ‘the similarity of each window between two groups ≥95%’. The unique genetic windows between MCB and the other three groups were visualized as similar genome regions using an online software ClicO FS v2.0.0 (http://codoncloud.com:3000/) (Cheong *et al*., 2015).

### Selective sweep analysis

Selective sweep analysis was conducted by measuring the patterns of allele frequencies in each 500‐kb fragment in 250‐kb steps along chromosomes, using the SNV data of QK and MCB (Table S2). Genomic regions under selective sweeps were measured by the fixation index (*F*_ST_) using VCFtools v0.1.13 (Danecek *et al*., 2011). Genomic regions with *F*_ST_ values >0.65 (about 5% top *F*_ST_ windows) were considered under strong selective sweeps.

### Data deposition

The reference genome sequence and annotation file of *qingke* (*cv*. Zangqing 320) are available at http://www.ibgs.zju.edu.cn/ZJU_barleygenome.htm. Raw reads were deposited in the Sequence Read Archive of NCBI (www.ncbi.nlm.nih.gov), and accession numbers are listed in Tables S1 and S2.

## Conflict of interest

The authors declare no conflict of interest.

## Supporting information


**Figure S1** Phylogenetic trees of 39 barley genotypes based on the 125 157 SNVs data set randomly distributed in the seven barley chromosomes.
**Figure S2** Population structure analysis of 39 barley genotypes.
**Figure S3** Genomic similarity analysis between *qingke* (QK) and the other three barley groups (a), or wild barley from Tibet (Wb‐T) and wild barley from the Near East (Wb‐NE) (b).
**Figure S4** Gene Ontology (GO) analysis of the 3675 and 4877 specific genes in the reference genome of Morex and Zangqing320, respectively.
**Figure S5** Unique Gene Ontology (GO) terms in Morex and Zangqing320 held by at least four specific genes.
**Figure S6** Single nucleotide variant (SNV) density of Igri and Barke along the reference genome of hulless barley *cv*. Zangqing320.
**Table S1** Summary of Zangqing320 WGS sequencing data.
**Table S2** Mapping RNA‐Seq reads of 39 samples to the genome of a hulless barley *cv*. Zangqing320 from the Tibetan Plateau.
**Table S3** Number of SNVs and CpG islands.
**Table S4** Genomic similarity analysis among the four barley groups based on unique genetic windows.

**Data S1** Physical position and annotation of the 2544 genes in selection sweep regions of *qingke* (QK) and modern cultivated barley (MCB).
