# Supplementary material for: Assembly and analysis of a qingke reference genome demonstrate its close genetic relation to modern cultivated barley
Source: Plant Biotechnol J. 2017 Oct 5;16(3):760–70. doi: 10.1111/pbi.12826 (PMC5814578; doi:10.1111/pbi.12826)
Supplement: Supplementary file 1 — Figure S1 Phylogenetic trees of 39 barley genotypes based on the 125 157 SNVs data set randomly distributed in the seven barley chromosomes. Figure S2 Population structure analysis of 39 barley genotypes. Figure S3 Genomic similarity analysis between qingke (QK) and the other three barley groups (a), or wild barley from Tibet (Wb‐T) and wild barley from the Near East (Wb‐NE) (b). Figure S4 Gene Ontology (GO) analysis of the 3675 and 4877 specific genes in the reference genome of Morex and Zangqing320, respectively. Figure S5 Unique Gene Ontology (GO) terms in Morex and Zangqing320 held by at least four specific genes. Figure S6 Single nucleotide variant (SNV) density of Igri and Barke along the reference genome of hulless barley cv. Zangqing320. Table S1 Summary of Zangqing320 WGS sequencing data. Table S2 Mapping RNA‐Seq reads of 39 samples to the genome of a hulless barley cv. Zangqing320 from the Tibetan Plateau. Table S3 Number of SNVs and CpG islands. Table S4 Genomic similarity analysis among the four barley groups based on unique genetic windows. [file PBI-16-760-s001.docx]

**Table S1.** Summary of Zangqing320 WGS sequencing data.

| Library Type | Platform | Size | Read Length | Bases (Gb) | Data accession no. |
| --- | --- | --- | --- | --- | --- |
| paired end | Illumina Hiseq 2500 | 250 bp | 2×125 bp | 32.37 | SRX1556764 |
| paired end | Illumina Hiseq 2500 | 300 bp | 2×125 bp | 84.63 | SRX1556764 |
| paired end | Illumina Nextseq 500 | 500 bp | 2×150 bp | 17.12 | SRX1556764 |
| mate pair | Illumina Hiseq 2000 | 2 kb | 2×100 bp | 117.40 | SRX1558327 |
| mate pair | Illumina Hiseq 2000 | 6 kb | 2×100 bp | 18.29 | SRX1558328 |
| - | PacBio SMRT sequencing | - | 3 kb^1^ | 38.46 | SRX1556762 |

^1^the average length of PacBio reads.

**Table S2.** Mapping RNA-Seq reads of 39 samples to the genome of a hulless barley *cv*. Zangqing320 from the Tibetan Plateau.

| Group | Sample  name/line | Growth habit | Data accession | Raw data and mapping statistics | | | | | |  | Mapped genes, transcripts, and SNV statistics | | |
| --- | --- | --- | --- | --- | --- | --- | --- | --- | --- | --- | --- | --- | --- |
|  |  |  |  | Raw reads | Raw bases (Gb) | Clean reads | Clean bases (%) | Mapped reads (%) | Reads mapped in proper pairs |  | Mapped genes | Mapped transcripts | Number of SNVs |
| MCB^1^ | Yiwuerleng | China | SAMN02483506^2^ | 24,481,894 | 2.45 | 19,930,844 | 78.23 | 84.57 | 13,322,196 |  | 21,898 | 25,253 | 90,840 |
|  | TX9425 | China | SAMN02483507^2^ | 19,920,328 | 1.99 | 16,604,974 | 80.32 | 98.17 | 13,655,282 |  | 21,538 | 24,542 | 81,134 |
|  | Esterel | France | SAMN02483510^2^ | 27,158,374 | 2.72 | 22,741,462 | 80.71 | 92.92 | 18,077,946 |  | 23,978 | 28,044 | 114,381 |
|  | Franka | Germany | SAMN02483511^2^ | 27,676,232 | 2.77 | 23,339,884 | 81.32 | 90.36 | 17,789,606 |  | 23,886 | 28,040 | 115,540 |
|  | Alexis | Denmark | SAMN02483509^2^ | 27,441,870 | 2.74 | 22,852,892 | 80.31 | 92.03 | 18,153,212 |  | 24,130 | 28,647 | 120,415 |
|  | Amagi Nijo | Japan | SAMN02483508^2^ | 25,548,686 | 2.55 | 20,959,274 | 78.74 | 97.85 | 17,663,748 |  | 24,162 | 27,656 | 106,456 |
|  | Nure | Italy | SAMN03952824^3^ | 56,731,418 | 5.67 | 47,485,984 | 79.89 | 96.55 | 38,730,632 |  | 30,642 | 37,573 | 181,212 |
|  | Tremois | French | SAMN03952823^3^ | 56,186,104 | 5.62 | 47,559,076 | 80.84 | 98.28 | 39,714,856 |  | 29,924 | 37,092 | 182,660 |
|  | Triumph | England | SRS1271890 | 55,978,672 | 5.60 | 47,248,774 | 80.69 | 95.31 | 36,521,846 |  | 28,971 | 34,984 | 149,959 |
| QK^1^ | Padanggamu | China | SAMN02483503^2^ | 26,515,802 | 2.65 | 22,092,040 | 80.13 | 98.25 | 18,160,474 |  | 23,168 | 26,627 | 60,737 |
|  | Beiqing5 | China | SAMN02483504^2^ | 20,673,144 | 2.07 | 17,376,528 | 81.09 | 94.06 | 14,142,794 |  | 23,419 | 27,118 | 78,432 |
|  | Himala2 | China | SAMN02483505^2^ | 20,494,044 | 2.05 | 17,064,132 | 80.27 | 97.64 | 13,911,084 |  | 21,211 | 24,201 | 54,617 |
|  | ZDM06853 | China | SRS1271891 | 32,733,718 | 4.09 | 32,690,696 | 99.57 | 97.77 | 27,892,728 |  | 29,039 | 34,826 | 130,699 |
|  | ZDM07571 | China | SRS1271892 | 24,873,596 | 3.11 | 24,846,880 | 99.63 | 97.47 | 21,257,176 |  | 27,699 | 32,946 | 121,017 |
|  | ZDM07612 | China | SRS1271893 | 25,654,596 | 3.21 | 25,619,430 | 99.50 | 97.25 | 21,668,572 |  | 27,621 | 32,893 | 122,398 |
|  | ZDM07646 | China | SRS1271894 | 27,377,096 | 3.42 | 27,334,572 | 99.49 | 95.97 | 22,855,314 |  | 28,049 | 33,578 | 129,294 |
|  | ZDM07648 | China | SRS1271895 | 22,260,358 | 2.78 | 22,232,332 | 99.63 | 97.42 | 18,771,294 |  | 27,124 | 31,597 | 111,741 |
|  | ZDM07747 | China | SRS1271896 | 23,925,268 | 2.99 | 23,890,312 | 99.53 | 97.16 | 19,774,802 |  | 25,096 | 29,309 | 93,244 |
|  | ZDM08108 | China | SRS1271897 | 24,565,720 | 3.07 | 24,532,740 | 99.56 | 97.05 | 20,717,546 |  | 27,136 | 32,122 | 113,975 |
|  | ZDM08141 | China | SRS1271898 | 22,793,326 | 2.85 | 22,761,282 | 99.57 | 97.11 | 19,420,236 |  | 27,651 | 32,525 | 127,363 |
|  | ZDM08205 | China | SRS1271899 | 22,953,204 | 2.87 | 22,922,768 | 99.61 | 97.51 | 19,637,562 |  | 26,919 | 31,631 | 117,891 |
|  | ZDM08217 | China | SRS1271900 | 23,566,028 | 2.95 | 23,541,502 | 99.70 | 96.97 | 19,349,348 |  | 26,057 | 30,431 | 115,319 |
|  | ZDM08841 | China | SRS1271901 | 23,554,282 | 2.94 | 23,528,142 | 99.68 | 95.65 | 19,191,044 |  | 26,457 | 31,230 | 119,547 |
|  | ZDM08846 | China | SRS1271902 | 20,376,738 | 2.55 | 20,355,398 | 99.72 | 96.88 | 17,090,508 |  | 25,514 | 29,742 | 107,946 |
|  | ZDM04815 | China | SRS1271903 | 27,624,580 | 3.45 | 27,594,194 | 99.70 | 96.79 | 23,059,810 |  | 27,475 | 32,907 | 118,787 |
|  | ZDM04817 | China | SRS1271904 | 22,453,382 | 2.81 | 22,427,734 | 99.69 | 96.76 | 19,139,128 |  | 26,742 | 31,898 | 134,769 |
| Group | Sample | Growth habit | Data accession | Raw data and mapping statistics | | | | | |  | Mapped genes, transcripts, and SNV statistics | | |
|  |  |  |  | Raw reads | Raw bases (Gb) | Clean reads | Clean bases (%) | Mapped reads (%) | Reads mapped in proper pairs |  | Mapped genes | Mapped transcripts | Number of SNVs |
|  | ZDM04908 | China | SRS1271905 | 25,083,706 | 3.14 | 25,058,702 | 99.72 | 97.10 | 20,741,012 |  | 26,348 | 30,717 | 105,117 |
| Wb-T^1^ | XZ2 | China | SAMN02483491^2^ | 32,708,440 | 3.27 | 27,353,310 | 80.75 | 97.42 | 22,823,344 |  | 25,905 | 30,507 | 113,157 |
|  | XZ12 | China | SAMN02483492^2^ | 33,519,518 | 3.35 | 28,258,648 | 81.26 | 93.53 | 22,311,722 |  | 24,998 | 29,559 | 145,640 |
|  | XZ15 | China | SAMN02483493^2^ | 31,176,332 | 3.12 | 26,331,292 | 81.39 | 90.28 | 20,003,266 |  | 24,973 | 29,445 | 120,547 |
|  | XZ21 | China | SAMN02483494^2^ | 31,092,152 | 3.11 | 26,429,186 | 82.05 | 97.45 | 22,252,854 |  | 24,710 | 29,256 | 122,172 |
|  | XZ174 | China | SAMN02483495^2^ | 38,150,502 | 3.82 | 31,970,376 | 80.80 | 88.29 | 23,896,302 |  | 25,496 | 30,228 | 129,511 |
|  | XZ181 | China | SAMN02483496^2^ | 38,614,144 | 3.86 | 32,524,982 | 81.18 | 88.66 | 24,157,692 |  | 27,065 | 32,000 | 150,611 |
| Wb-NE^1^ | ECI-2-0 | Israel | SAMN02483497^2^ | 35,912,464 | 3.59 | 30,811,558 | 82.84 | 92.11 | 23,803,654 |  | 26,075 | 30,971 | 173,372 |
|  | Tabigha-B-63 | Israel | SAMN02483501^2^ | 42,515,554 | 4.25 | 36,336,986 | 82.53 | 97.14 | 29,752,128 |  | 27,046 | 32,826 | 178,371 |
|  | Iran-6-26 | Iran | SAMN02483499^2^ | 34,901,052 | 3.49 | 29,637,564 | 82.03 | 96.50 | 25,001,966 |  | 26,417 | 31,848 | 160,549 |
|  | Turkey-19-24 | Turkey | SAMN02483500^2^ | 38,820,072 | 3.88 | 32,703,704 | 81.15 | 90.54 | 25,026,386 |  | 27,951 | 33,665 | 184,000 |
|  | ECI-6-0 | Israel | SAMN02483498^2^ | 27,071,504 | 2.71 | 23,396,946 | 83.78 | 95.58 | 19,205,682 |  | 24,318 | 28,785 | 146,013 |
|  | Tabigha-T-0 | Israel | SAMN02483502^2^ | 30,780,278 | 3.08 | 25,734,288 | 80.46 | 96.03 | 21,244,010 |  | 24,467 | 29,019 | 149,622 |

^1^ MCB: Modern Cultivated Barley, QK: *qingke*-hulless barley from the Tibetan Plateau, Wb-T: wild barley from the Tibetan Plateau, Wb-NE: wild barley from the Near East. ^2^ RNA-Seq data adapted from Dai *et al*. (*Proc Natl Acad Sci U S A,* 2014, 111: 13403-13408). ^3^ RNA-Seq data adapted from Wang *et al*. (*Front Plant Sci*, 2016, 7).

**Table S3.** Number of SNVs and CpG island between Morex and Zangqing320.

| Structure | Reference | Number | | | | | | | |
| --- | --- | --- | --- | --- | --- | --- | --- | --- | --- |
|  |  | 1H | 2H | 3H | 4H | 5H | 6H | 7H | Total |
| SNVs | Morex^1^ | 481,684 | 807,062 | 1,068,871 | 1,056,302 | 1,076,618 | 947,405 | 615,503 | 6,053,445 |
|  | Zangqing320^2^ | 141,053 | 192,812 | 187,682 | 121,510 | 182,701 | 146,441 | 175,807 | 1,148,006 |
| CpG islands | Morex | 24,488 | 33,905 | 33,928 | 28,943 | 31,887 | 25,467 | 27,431 | 206,049 |
|  | Zangqing320 | 24,502 | 34,003 | 33,932 | 28,890 | 31,913 | 25,442 | 27,470 | 206,152 |

^1^ SNVs are called after mapping in-depth genome re-sequencing reads of Zangqing320 to the reference genome of Morex (Mascher *et al.,* 2017). ^2^ SNVs are called by mapping RNA-Seq reads of the 39 barley genotypes to the reference genome of Zangqing320.

**Table S4.** Genomic similarity analysis among the four barley groups based on unique genetic windows.

| Groups | Chromosome^1^ | | | | | | | |
| --- | --- | --- | --- | --- | --- | --- | --- | --- |
|  | 1H | 2H | 3H | 4H | 5H | 6H | 7H | Total |
| MCB & QK | 54 (60.67%) | 65 (59.63%) | 45 (55.56%) | 30 (49.18%) | 47 (35.61%) | 25 (30.86%) | 68 (42.24%) | 334 (46.78%) |
| MCB & Wb-T | 20 (22.47%) | 18 (16.51%) | 16 (19.75%) | 23 (37.70%) | 47 (35.61%) | 34 (41.98%) | 68 (42.24%) | 226 (31.65%) |
| MCB & Wb-NE | 15 (16.85%) | 26 (23.85%) | 20 (24.69%) | 8 (13.11%) | 38 (28.79%) | 22 (27.16%) | 25 (15.53%) | 154 (21.57%) |
| QK & MCB | 53 (49.53%) | 67 (59.82%) | 43 (29.05%) | 27 (22.50%) | 41 (19.71%) | 26 (30.23%) | 62 (46.97%) | 319 (34.94%) |
| QK & Wb-T | 44 (41.12%) | 25 (22.32%) | 96 (64.86%) | 85 (70.83%) | 153 (73.56%) | 49 (56.98%) | 56 (42.42%) | 508 (55.64%) |
| QK & Wb-NE | 10 (9.35%) | 20 (17.86%) | 9 (6.08%) | 8 (6.67%) | 14 (6.73%) | 11 (12.79%) | 14 (10.61%) | 86 (9.42%) |
| QK & Wb-T | 59 (78.67%) | 41 (65.08%) | 117 (90.70%) | 107 (92.24%) | 187 (91.67%) | 59 (75.64%) | 101 (81.45%) | 671 (85.04%) |
| QK & Wb-NE | 16 (21.33%) | 22 (34.92%) | 12 (9.30%) | 9 (7.76%) | 17 (8.33%) | 19 (24.36%) | 23 (18.55%) | 118 (14.96%) |

^1^The length of chromosome referred to the genome of Zangqing320. Digits show the number of unique genetic windows between every two groups, while percentages in the parentheses show genomic similarities calculated based on the total length of unique windows, excluding the overlap of slide windows. MCB, Modern Cultivated Barley; QK, *qingke*, hulless barley from the Tibetan Plateau; Wb-T, wild barley from the Tibetan Plateau; Wb-NE, wild barley from the Near East.


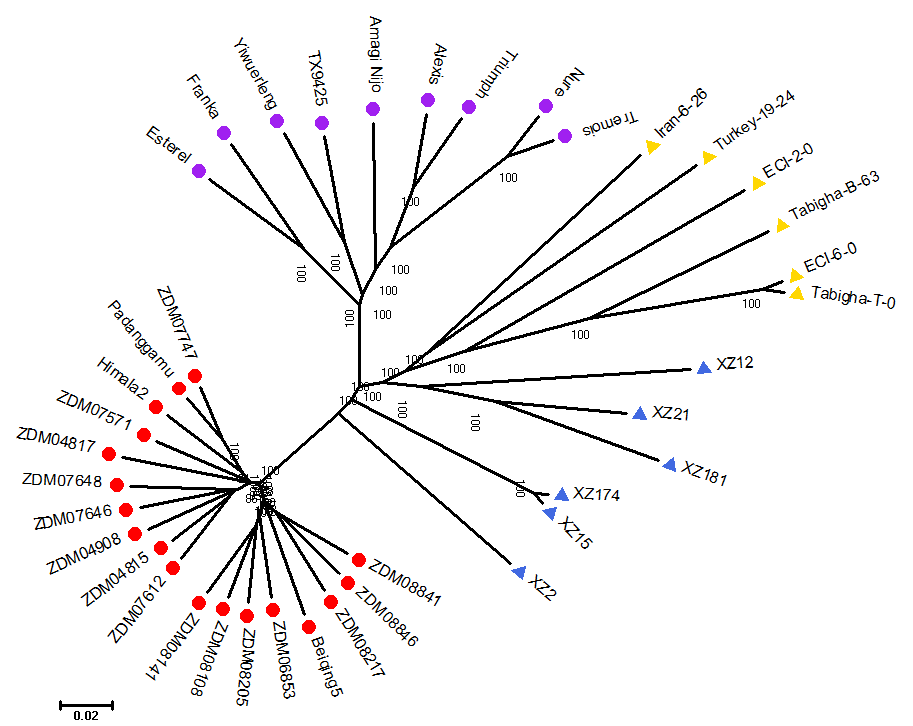


**Figure S1.** Phylogenetic trees of 39 barley genotypes based on the 125,157 SNVs dataset randomly distributed in the seven barley chromosomes. The neighbor-joining method was used with 1,000 bootstraps. Branches marked by red and purple dots represent *qingke* and modern cultivated barley, while those marked by blue and yellow triangles represent wild barley from the Tibetan Plateau and Near East, respectively.

**Figure S2.** Population structure analysis of 39 barley genotypes. Each color denotes one population, and each vertical bar represents one accession. The percentages of contribution from the ancestral populations are indicated by the lengths of colored segments. The number of clusters (K) was set from 2 to 6. By evaluating K, a clear evolutionary divergence between *qingke*, modern cultivated barley and wild barley genotypes was found with K=3. When K=4, genotypes from the Tibetan Plateau fell into a new subgroup within the wild barley population. When K=5, wild barley from the Near East were divided into two groups.

**Figure S3.** Genomic similarity analysis between *qingke* (QK) and the other three barley groups (A), or wild barley from Tibet (Wb-T) and wild barley from the Near East (Wb-NE) (B). The outer track of the circos diagram shows the seven chromosomes (1H–7H) of barley in each of the four groups: purple, modern cultivated barley (MCB); red, QK; blue, Wb-T; yellow, Wb-NE. The number on each chromosome indicates the genomic position on the genome of Zangqing320 (Mb). Similar blocks are connected with lines, and each line represents one unique window (500 kb) of the genome with the highest similarity between QK and other barley groups.

**Figure S4.** Gene Ontology (GO) analysis of the 3,675 and 4,877 specific genes in the reference genome of Morex and Zangqing320, respectively. (A) Common and unique GO terms of the 3,675 and 4,877 specific genes in Morex and Zangqing320. (B) Top 1% GO terms of the 3,675 specific genes in Morex. (C) Top 1% GO terms of the 4,877 specific genes in Zangqing320. P, Biological Process; F, Molecular Function; C, Cellular Component. Numbers of private and specific genes with a certain GO term are shown on the left and right edge of the bar, respectively.

**Figure S5.** Unique Gene Ontology (GO) terms in Morex and Zangqing320 held by at least four specific genes. Numbers of specific and private genes with a certain GO term are shown in parentheses separated by a comma.

**Figure S6.** Single nucleotide variant (SNV) density of Igri and Barke along the reference genome of hulless barley *cv.* Zangqing320. The three tracks (from outside to inside) are chromosomes of *cv*. Zangqing320, SNV density of Igri (light purple) and Barke (dark purple). The densities of SNV are represented by the number of non-overlapping 500 kb windows. Black triangles refer to genomic regions with low genetic diversity between Zangqing320 and Igri/Barke.
